# Left common iliac vein diameter in patients referred for lower limb venous duplex ultrasound

**DOI:** 10.1177/17085381231153540

**Published:** 2023-01-23

**Authors:** Damon Judges, Chen Liu, Sarah Onida, Tristan Robert Alexander Lane, Alun Huw Davies

**Affiliations:** 1 Department of Surgery and Cancer, 4615 Imperial College London, London, UK; 2 Cambridge Vascular Unit, Addenbrookes Hospital, Cambridge, UK

**Keywords:** Venous, vascular, ultrasound

## Abstract

**Introduction:**

Evidence regarding ultrasound assessment of left common iliac vein diameter (LCIV) is limited. Extensive work is currently being undertaken worldwide on non-thrombotic iliac vein lesions to identify patients who may benefit from intervention to alleviate symptoms of chronic venous obstruction. Interventions include long-term stent implantation to improve vein diameter stenosis. This study aimed to assess a cohort of symptomatic venous patients and the diameter of the LCIV in these patients.

**Methods:**

: Retrospective medical records review of all patients attending a tertiary vascular surgery centre who underwent a venous duplex ultrasound assessment between April 2017 and February 2018 were analysed for assessment of LCIV. Medical records of those patients with documented LCIV diameter were assessed over 18 months of follow-up.

**Results:**

: A total of 672 (271 males, 401 females) LCIV diameter measurements were collected. The age of the patients ranged from 21 to 95 years (mean = 56.38). Median LCIV diameter overall was 7.64 mm (IQR 5.80mm–9.00 mm). 40 patients (6%) were reported to have a LCIV diameter measurement of < 4 mm, 8 (20%) male and 32 female (80%). 17 of these 40 patients (47.5%) were treated conservatively. Median LCIV diameter was 3.4 mm (IQR 2.5–3.7). 21 of these 40 patients (52.5%) underwent superficial venous intervention only, with a median LCIV diameter of 3.5 mm (IQR 3.2–3.7) and 2 out of these 40 patients (5%) underwent deep venous stenting (2/2 female – 100%), with a median LCIV diameter of 2.9 (IQR 2.9–2.9). No patients underwent both superficial and deep venous treatment in this 40 patient cohort. In those undergoing superficial venous intervention, 4 (19%) underwent repeat treatment. The two deep venous stenting patients underwent magnetic resonance venogram and venogram with intravascular ultrasound to allow stent placement, which confirmed a narrowed left common iliac vein. Primary stent patency at 18 months was 100%.

**Conclusion:**

In this large study cohort of venous duplex assessments the median vein diameter was 7.64 mm and 40 patients out of 672 had a vein diameter smaller than 4 mm. 2 patients underwent deep venous stenting with primary patency of 100%.

## Introduction

Non-thrombotic iliac veins lesions (NIVL) such as iliac vein compression syndrome (formerly known as May–Thurner syndrome) or extrinsic compression from other sources can contribute to the pathophysiology of chronic venous disease in the lower limbs.^1–4^ Endovascular interventions for the management of NIVL have been increasing over recent years; however, the evidence surrounding the criteria for intervention remains limited.^5^ Furthermore, the evidence around the prevalence of NIVL in the general population and the clinical significance on asymptomatic patients as well as those with lower limb venous insufficiency is still evolving.^6–9^

Earlier studies have utilised indirect methods or computer tomography venography to assess vein diameter.^10,11^ The aim of this paper is to develop a better understanding of the left common iliac vein (LCIV) anatomy in patients with symptoms of venous insufficiency and contribute to the evidence base surrounding the diagnosis and management of patients with NIVL.

## Methods

This study was a retrospective review of duplex ultrasound reports from April 2017 to February 2018 that documented a measurement (mm) of the LCIV diameter for patients referred for assessment of lower limb venous insufficiency. Data was extracted from electronic patient records in August 2020 providing 18 months minimum clinical follow-up.

### Institutional approval

The study was classed as service evaluation according to the NHS Research Ethics Service – analysis of routinely collected data and therefore formal ethical review and informed patient consent was not necessary.

### Patients

All patients were referred to the Vascular surgery department at Imperial College Healthcare NHS Trust and underwent lower limb venous duplex to investigate symptoms of lower limb venous insufficiency in either lower limb (CEAP 2–6).

### Duplex Ultrasound Protocol

The department’s venous insufficiency protocol was adapted to include the assessment of the ilio-caval vessels and measurement of the LCIV. At the completion of a standing lower limb venous insufficiency venous duplex patients were asked to lie supine and a check of ilio-caval venous patency was performed in addition to a transverse diameter measurement (mm) of the LCIV as the right common iliac artery crosses the LCIV. The results were reported on the standard lower limb venous duplex report. The research protocol was discussed at length at Vascular scientist education meetings and initial measurements were recorded with two scientists in the room to ensure a standardised measurement and minimise inter-operator variation.

### Ultrasound machine

Duplex ultrasound was performed by the Trust`s experienced vascular scientists using a C 1–5 MHz curved array transducer on a Phillips Epic 7, IU22 or GE logic E9 ultrasound machine.

### Statistics

Data was assessed visually for normality and formally assessed with the Shapiro-Wilk test. Parametric data is presented as mean and standard deviation, non-parametric data is presented as median and interquartile range. Statistical analysis of the sample was performed using STATA (version 16, STATACORP, College Station, Texas, USA), JMP Pro 15 (SAS, Cary, North Carolina, USA) and Wizard 2 (Evan Miller, Chicago, Illinois, USA).

## Results

**Figure 1.**
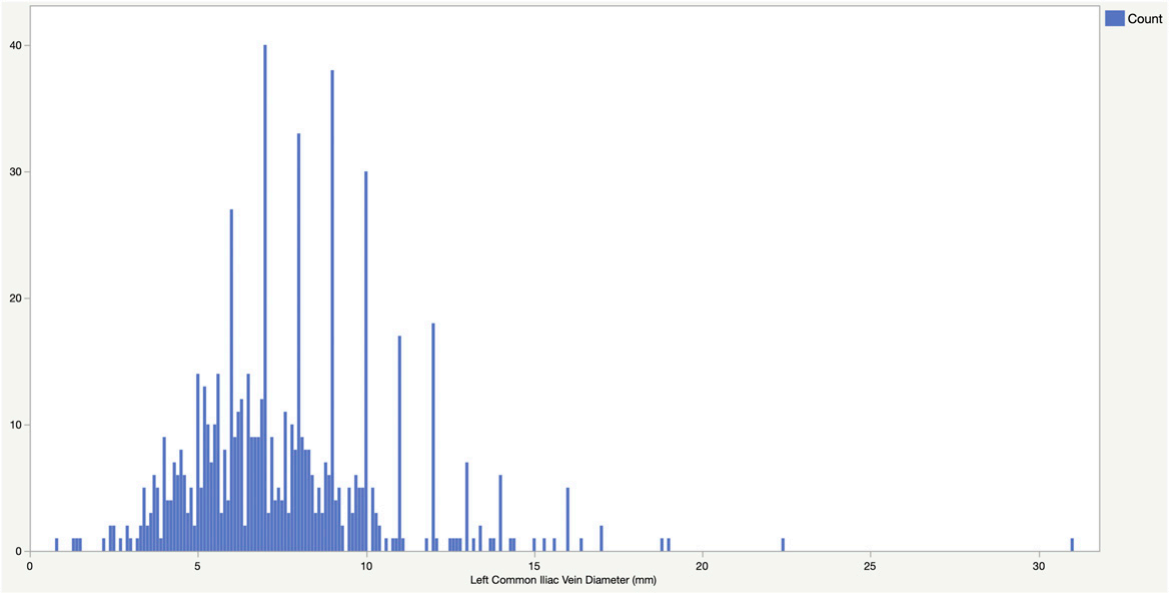
Distribution of Left Common Iliac Vein diameter (mm).

**Figure 2.**
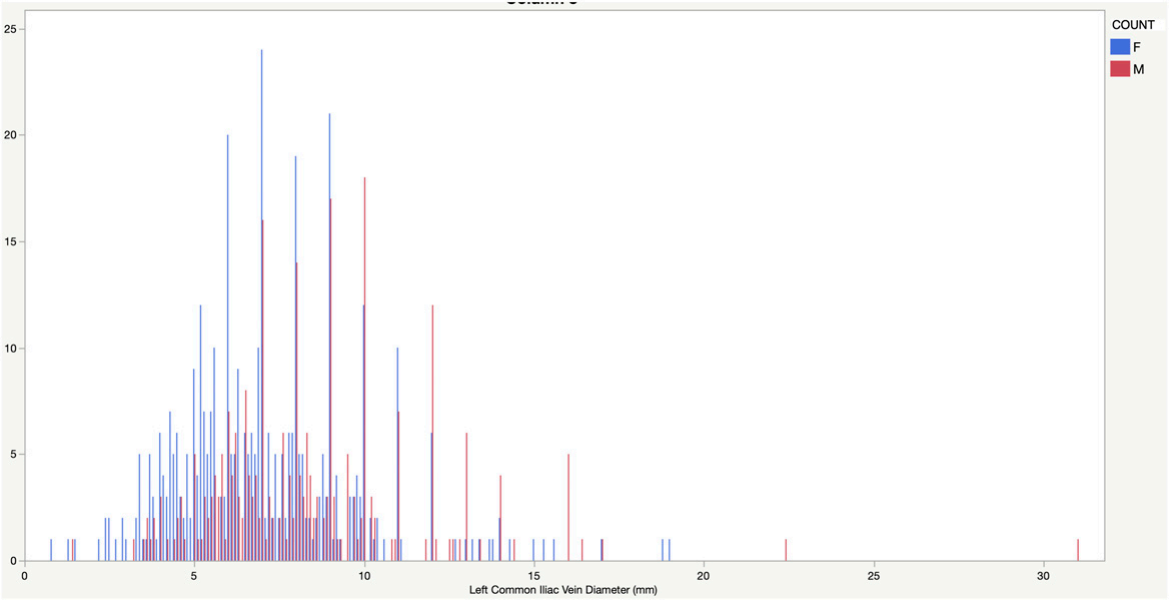
Distribution of Left Common Iliac Vein diameter (mm) stratified by sex (M - Male – Red, F - Female – Blue).

Between April 2017 and February 2018 (11 months), a total of 1249 lower limb venous duplex ultrasound scans were performed, and then reviewed for this study. This number comprises a range of venous examinations and only those patients referred for assessment of venous insufficiency were included in the results. A total of 672 (271 males, 401 females) LCIV diameter measurements were collected. The age of the patients ranged from 21 to 95 years (mean = 56.38). A summary of the findings is detailed in Table 1 below. LCIV diameter was significantly greater in males (*p* < 0.001). Figure 1 shows the entire distribution and Figure 2 shows the male and female distribution.

**Table 1.** Min, Max, Mean, standard deviation, Median and Interquartile Range.

	Mean	Standard deviation	Median	Interquartile range	Min (mm)	Max (mm)	*n* =
Total	7.64	2.92	7.10	5.80–9.00	0.80	31.00	672
Males	8.42	3.16	8.00	6.40–10.00	1.40	31.00	271
Females	7.12	2.63	6.90	5.30–8.70	0.80	19.00	401

### Left common iliac vein diameter <4 mm:

Of the 672 patients scanned, 40 patients (6%) were reported to have an LCIV diameter measurement of < 4 mm, 8 (20%) male and 32 female (80%). All patients presented with symptoms of leg swelling or symptomatic varicose veins. Three patients had a previous history of DVT with evidence on duplex ultrasound and 2 patients were found to have an acute DVT at the time of scanning.

Of these 40 patients, 17 patients (47.5%) were treated conservatively (4/17 male – 24%), with 3 advised to wear compression hosiery long-term. Median LCIV diameter was 3.4 mm (IQR 2.5–3.7, Min 1.4 Max 3.9). 21 patients (52.5%) underwent superficial venous intervention only (4/21 male – 19%), with a median LCIV diameter of 3.5 mm (IQR 3.2–3.7, Min 0.8 Max 3.8).

Two patients (5%) underwent deep venous stenting (2/2 female – 100%), with a median LCIV diameter of 2.9 (IQR 2.9–2.9, Min 2.9 Max 2.9). There was no history of DVT in the stenting patients. No patients underwent both superficial and deep venous treatment in this cohort.

In those undergoing superficial venous intervention, 4 (19%) underwent repeat treatment – 2 cases of repeat foam sclerotherapy, 1 case of foam sclerotherapy after radiofrequency ablation to residual varicosities and 1 case of redo saphenofemoral ligation and multiple phlebectomies after initial phlebectomies only.

Thirteen patients (33%) underwent further imaging; 3 (17.6%) of the conservatively managed patients underwent further imaging, and 8 (38%) of the patients undergoing superficial intervention underwent further imaging, though 7 patients underwent repeat confirmatory duplex ultrasound prior to treatment. The eighth patient underwent a magnetic resonance venogram prior to multiple phlebectomies.

The two deep venous stenting patients underwent magnetic resonance venogram and venogram with intravascular ultrasound to allow stent placement, which confirmed a narrowed left common iliac vein. Primary stent patency at 18 months was 100% (2/2).

## Discussion

This single centre study assessed 672 patients and found the mean LCIV diameter was 7.64 mm; this was larger in males (8.42 mm) than females (7.12 mm). There is a limited body of evidence with which to compare this study’s mean LCIV diameters. A previous study compared LCIV diameters of female patients with lower limb DVT (*n* = 21) and an age matched control group (*n* = 26) who reported to Emergency Departments with abdominal pain and reported mean LCIV diameters of 4.0 mm and 6.5 mm, respectively.^12^ Another study reported on patients with DVT caused by iliac vein compression with mean LCIV diameters of 3.5 mm (DVT group, *n* = 10 (5 males, 5 females)) and 11.5 mm (control group, *n* = 14).^13^

However, in this study, only 5 patients had an acute or chronic DVT and 2 other patients underwent deep venous stenting intervention. In addition, the reported severity of symptoms and duplex results varied drastically between patients (not quantitatively analysed in this cohort).

This study shows that, as found in the disparate literature, vein diameter is not always associated with treatment necessity.^14–17^ Only 6% of patients in this cohort had a < 4 mm LCIV, and of those only 2 (0.3% of total cohort) required deep venous intervention. This suggests that in an unselected cohort, the prevalence of clinically significant LCIV stenosis is low.

Additionally, those patients with small LCIV who underwent intervention responded well out to 18 month follow-up, with 1 only one true treatment failure (6%) – following superficial venous intervention. However, the numbers in this study preclude any firm judgements regarding longevity of intervention.

These findings are complementary to a recent study that found a high percentage of asymptomatic healthy volunteers showed signs of significant iliac vein compression on venography.^6^ Previous studies have even suggested left iliac vein compression as an anatomical variant instead of pathology,^7^ and other studies found significant differences in diameter in the standing and lying positions.^18,19^ These results emphasise the need for a detailed understanding of population characteristics relating to NIVL and the importance of detailed clinical examination and evaluation of these patients.

However, work by Arendt et al.,^10^ assessing vein segments using centreline adjusted CT venography, found significantly larger vein diameters in the region of the LCIV. Their findings, in a different modality with post-processing of the imaging, showed a mean LCIV of 12.2 mm, compared to this study’s 7.64 mm. This difference may be due to different modalities, demographics or the fact that Arendt et al’s study only included post-thrombotic patients (albeit only healthy segments were assessed).

Duplex ultrasound is widely recognised as not having the sensitivity to enable diagnosis of NIVL as a single imaging modality.^4,6,20^ However, the role of duplex ultrasound (with a well-trained operator) in the diagnosis, treatment and post-operative surveillance of NIVL in conjunction with venography, computer tomography venography and intravascular ultrasound should not be underestimated.

### Limitations

The actual number of lower limb venous duplex scans during April 2017–February 2018 was 1249, this included pre-op vein mapping, post op varicose vein ablation/patency, post op iliac stent, ad-hoc DVT scans for other research projects and other miscellaneous venous scans. There was no way to retrospectively differentiate these scans to quantify the success rate of obtaining a LCIV diameter measurement, however all vascular scientists in the department reported high confidence in obtaining a LCIV measurement according to the scan protocol in the vast majority of patients sent for lower limb venous insufficiency assessment. It is hoped future studies will help confirm this.

The small number of patients in this study with <4.0 mm LCIV limits the generalisability of the findings in the study.

As this study was a retrospective review of duplex ultrasound scan results, there was no gold standard diagnostic comparator such as venography performed to compare the measurements with. Comparison with other studies assessing NIVL with venography and IVUS was also limited due to the majority of studies reporting in percentage of compression rather than diameter of the vessel.^4,6–9^

## Conclusion

This study provides one of the largest samples to date, to describe the LCIV diameter characteristics of patients with symptoms of venous insufficiency as assessed by duplex ultrasound. In 672 patients, median LCIV was 7.64 mm with 40 patients having an LCIV <4 mm.
